# SIG1, a Sigma Factor for the Chloroplast RNA Polymerase, Differently Associates with Multiple DNA Regions in the Chloroplast Chromosomes *in Vivo*

**DOI:** 10.3390/ijms131012182

**Published:** 2012-09-25

**Authors:** Mitsumasa Hanaoka, Maiko Kato, Misato Anma, Kan Tanaka

**Affiliations:** 1 Division of Applied Biological Chemistry, Graduate School of Horticulture, Chiba University, Matsudo 648, Matsudo, Chiba 271-8510, Japan; E-Mails: mai5_a8s@yahoo.co.jp (M.K.); z9h2002@students.chiba-u.jp (M.A.); 2 Division of Bioresources, Chemical Resources Laboratory, Tokyo Institute of Technology, Nagatsuta 4259-R1-29, Midori-Ku, Yokohama 226-8503, Japan; E-Mail: kntanaka@res.titech.ac.jp

**Keywords:** chloroplast, chromatin immunoprecipitation, transcriptional regulation, sigma factor, SIG1, high-light stress, *Arabidopsis thaliana*

## Abstract

Chloroplasts have their own DNA and gene expression systems. Transcription in chloroplasts is regulated by two types of RNA polymerase, nuclear-encoded plastid RNA polymerase (NEP) and plastid-encoded plastid RNA polymerase (PEP), and multiple sigma factors for PEP. To study transcriptional regulation in chloroplasts, a molecular genetic approach has extensively been used. However, this method may include indirect effects, and it cannot be applied to the analysis of factors essential to survival. These limitations make understanding specific regulation by transcription factors difficult. Chromatin immunoprecipitation (ChIP) is a powerful and useful tool for obtaining information on transcription-factor binding sites; it can directly detect dynamic changes in their interaction patterns *in vivo*. To further understand transcriptional regulation in chloroplasts, we here established a ChIP-based method in *Arabidopsis thaliana* and analyzed the binding pattern of a chloroplast sigma factor, SIG1. We found that SIG1 specifically binds to newly identified target promoters as well as to a set of promoters of genes whose mRNA expression is dependent on OsSIG1 in rice and that this binding changed in response to high-light stress. These results suggested that the ChIP-based approach is very useful in understanding transcriptional regulation of chloroplast genes and can overcome several problems posed by conventional methods.

## 1. Introduction

Chloroplasts are plant organelles that originated from an endosymbiotic event involving an ancestral oxygen-evolving photosynthetic cyanobacterium. Chloroplasts thus have their own genomes and transcription-translation machineries of cyanobacterial origin. The chloroplast genomes of most higher plants are circular, double-stranded DNA molecules containing about 120 genes required for photosynthesis, gene expression, and some metabolic functions [1,2]. However, these genes encode only some of the chloroplast proteins; most other proteins required for chloroplast functions are encoded by the nuclear genome [3]. Thus, coordinated expression of nuclear and chloroplast genes is essential for normal chloroplast biogenesis and development [4].

In the chloroplasts of higher plants, there are at least two types of plastid RNA polymerase, called NEP (nuclear-encoded plastid RNA polymerase) and PEP (plastid-encoded plastid RNA polymerase) [5,6]. The NEP is a T7 bacteriophage-type, single-subunit enzyme related to mitochondrial RNA polymerase and is mainly involved in the transcription of non-photosynthetic housekeeping genes [7,8]. However, PEP is a eubacteria-type, multi-subunit enzyme that is required to transcribe photosynthesis-related genes [9,10]. Genes encoding core subunits of PEP (*rpoA*, *B*, *C1* and *C2*) are located in the chloroplast genome, but those encoding sigma factors, which are required for promoter recognition and transcription initiation, are located in the nuclear genome [11]. In *Arabidopsis thaliana*, six genes encode PEP sigma factors (*SIG1*–*SIG6*) and specific roles for each sigma factor in response to various developmental and/or environmental conditions have been clarified [12–16].

To study the function of each sigma factor in transcriptional regulation of chloroplast genes, biochemical as well as molecular genetic approaches have mainly been used. Biochemical studies, such as gel-shift analyses and *in vitro* transcription assays, have clearly demonstrated specific interactions between promoter DNA and sigma factor protein(s) [17,18]. In addition, molecular genetic analyses using knockout mutants and various transformants have identified specific target genes for each sigma factor [19–23]. For example, SIG2 and SIG6 have been found to be cooperatively involved in light-dependent chloroplast development, but with different target genes; SIG2 targets a group of tRNA genes, while SIG6 targets a wide range of photosynthesis genes that function specifically in early chloroplast differentiation [22–26]. On the other hand, SIG5 has been identified as a sigma factor that responds to blue light as well as multiple abiotic stresses, such as intense light, salt, and cold, to regulate a very limited number of targets, including *psbD* BLRP (blue-light responsive promoter) [21,27]. However, biochemical approaches cannot demonstrate these interactions under physiological conditions, and genetic approaches may include indirect effects, so understanding specific regulation by the protein of interest is difficult. Furthermore, knockout mutants of genes encoding proteins that are essential for survival are impossible to obtain. Thus, a novel approach is required to overcome these problems.

Chromatin immunoprecipitation (ChIP) is a recently-established and convenient tool for obtaining information on target regions of DNA-binding proteins. This method can directly detect dynamic changes in interaction patterns *in vivo* [28]. Initially, this method was designed to detect specific chromatin modifications [29] and was later used to identify transcription factor binding sites. ChIP analysis involves cross-linking cellular DNA-protein complexes with formaldehyde, cell disruption, and sonication of the crude extracts for DNA fragmentation (to ~500–600 bp on average). Then, immunoprecipitation with a specific antibody is performed to purify the protein of interest, together with cross-linked target DNA. Finally, cross-links are removed with heat treatment, and DNA is purified. Consequently, the genomic regions bound to the protein of interest at the moment of formaldehyde cross-linking can be specifically enriched. Finally, the levels of purified genomic regions can be measured by quantitative PCR (qPCR) and their signals analyzed as the percent recovery against the amount of input DNA.

In this work, we used a ChIP-based method to further investigate the transcriptional regulation of chloroplast genes in *A. thaliana*. We analyzed the binding pattern of SIG1, one of the sigma factors for PEP. We identified specific interactions between SIG1 and several chloroplast promoters and found that binding decreased in response to high-light stress. We concluded that ChIP analysis is useful to understand transcriptional regulation of chloroplast genes and can detect dynamic changes in DNA-protein interactions in chloroplasts *in vivo*.

## 2. Results

### 2.1. Accumulation of SIG1 Protein during Development of *A. thaliana* Seedlings

**Figure 1:**
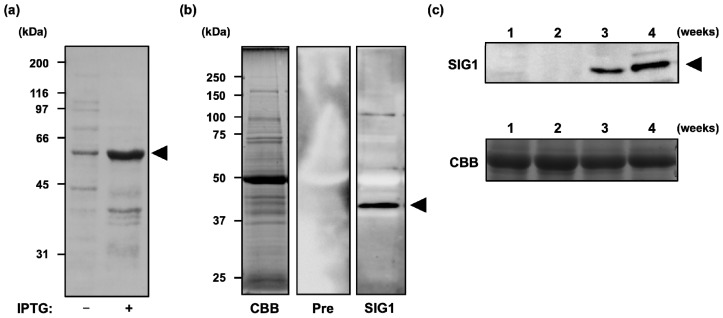
Western blot analysis of total proteins prepared from wild type (Col-0) *Arabidopsis thaliana* grown under continuous normal light (50 μmol photons m^−2^ s^−1^) and separated by 10% SDS-PAGE. (**a**) Expression of full-length SIG1 protein in *E.coli* before and after IPTG induction. The positions of molecular weight markers are indicated as kiloDaltons (kDa) on the left; (**b**) Specificity of SIG1 antibody. Total proteins (10 μg) from four-week-old plants were treated with SIG1 antibody (SIG1) or preimmune serum (Pre). (**c**) Levels of SIG1 protein during seedling development. Total proteins (20 μg) of plants grown for 1, 2, 3, and 4 weeks were analyzed using the SIG1 antibody (SIG1). CBB (Coomassie Brilliant Blue)-stained gels are shown as loading controls.

Successful ChIP analysis usually requires a specific antibody against the protein of interest. Therefore, we initially prepared a rabbit polyclonal antibody against a recombinant full-length SIG1 protein (502 amino acids) that was expressed in *E.coli* (Figure 1a) and purified as a His-tag protein. Antiserum specificity was checked by immunoblot analysis. Although it has been reported in a previous study that SIG1 detected around 50 kDa [18], major single band around 40 kDa, suggesting a mature protein of SIG1, could be detected in our experimental condition (Figure 1b), and we used this antibody for further immunoblot and ChIP analyses.

Before we performed ChIP analysis to identify SIG1-dependent promoters, the accumulation of SIG1 during seedling development was examined. *Arabidopsis thaliana* wild-type plants were cultivated under standard conditions and sampled after 1, 2, 3, and 4 weeks. As shown in Figure 1c, SIG1 protein accumulated gradually at later stages (3–4 weeks) of seedling development. Thus, the ChIP analysis was performed using four-week-old plants.

### 2.2. Identification of SIG1-Dependent Promoters in *A. thaliana* Chloroplasts

**Figure 2:**
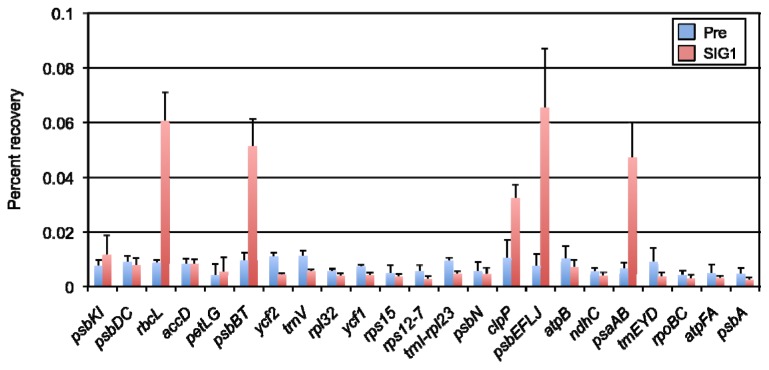
Chromatin immunoprecipitation (ChIP-qPCR) analysis was performed in the presence of preimmune serum or anti-SIG1 antibody using whole-cell extract prepared from wild type (Col-0) *Arabidopsis thaliana* grown under continuous normal light (50 μmol photons m^−2^ s^−1^). Levels of immunoprecipitated DNA of various promoter regions were calculated as percent recovery of the total input DNA. Data are means ± SD of three experiments.

To investigate target genes of SIG1, a chloroplast sigma factor in *A. thaliana*, we performed ChIP-qPCR analysis to detect the binding of SIG1 with its target promoters *in vivo*. Rosette leaves of wild-type *A. thaliana* plants grown in normal-light conditions for four weeks were treated with formaldehyde to fix DNA-protein interactions, whole-cell extracts containing total DNA-protein complexes were used for immunoprecipitation with the SIG1 antibody, then DNA was unlinked from the proteins. Purified DNA amounts were determined by quantitative PCR. To identify SIG1-dependent promoters across the chloroplast genome of *A. thaliana*, we designed 23 primer sets to amplify each upstream region of various chloroplast genes and quantified the binding levels of SIG1. In this method, the amount of purified DNA after immunoprecipitation reflected specific binding of a protein to target promoters. The amount of purified DNA was expressed as percent recovery against several dilutions of input DNA, which was plotted on standard curve obtained from qPCR analysis. Of 23 promoter regions, immunoprecipitated DNA could be detected for several specific promoters, *rbcL*, *psbBT*, *clpP*, *psbEFLJ*, and *psaAB* (Figure 2), suggesting that transcription from these promoters could be controlled by SIG1 in this condition. Other promoters, such as *accD*, *trnEYD*, and *psbN*, which were previously identified as NEP, SIG2, and SIG3-dependent promoters [8,19,22,24], seemed not to be recognized by SIG1.

### 2.3. High-Light-Dependent Release of SIG1 from Its Target Promoters

**Figure 3:**
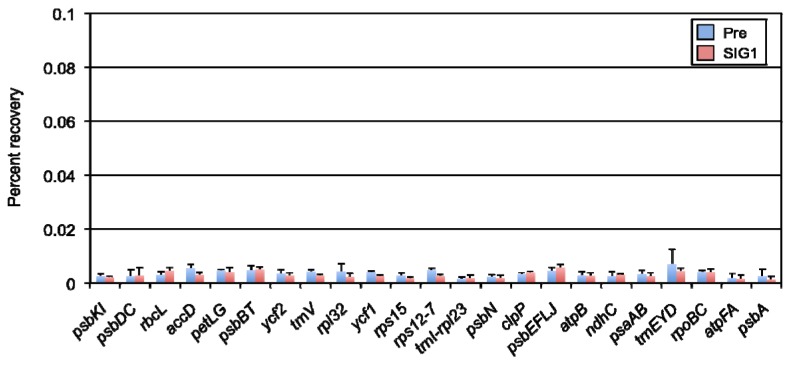
ChIP-qPCR analysis was performed in the presence of preimmune serum or anti-SIG1 antibody using whole-cell extract prepared from wild type (Col-0) *Arabidopsis thaliana* grown under high light (1,200 μmol photons m^–2^ s^–1^) for 1 h after continuous normal light (50 μmol photons m^–2^ s^–1^). The levels of immunoprecipitated DNA of various promoter regions were calculated as percent recovery of total input DNA. Data are means ± SD of three experiments.

An advantage of the ChIP method is to detect changes in binding between DNA and a protein of interest. Therefore, we examined dynamic changes in the SIG1 binding level to target promoters under high-light stress conditions. Whole-cell extracts were prepared from rosette leaves treated with high light (1,200 μmol photons m^−2^ s^−1^) for 1 h, and ChIP-qPCR analyses were performed. As shown in Figure 2, the basal SIG1 binding level to its target promoters under normal light was significant; SIG1 binding was dramatically decreased by high-light exposure (Figure 3). This binding pattern observed in all five promoters, while other promoters showed no major changes in binding activity. These results indicated that SIG1 binds strongly to target promoters under normal light conditions, and it can be released in response to high-light stress.

### 2.4. Accumulation of SIG1 during the Change in Light Conditions

**Figure 4:**
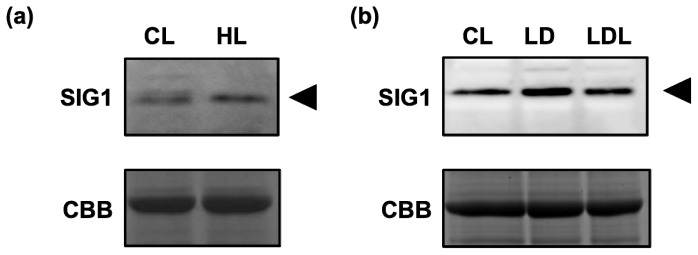
Protein levels of SIG1 under various light conditions. (**a**) *Arabidopsis thaliana* wild-type (Col-0) plants grown under continuous normal light (50 μmol photons m^−2^ s^−1^) for 4 weeks (CL) were shifted to high light (1,200 μmol photons m^−2^ s^−1^) for 1 h (HL). Total proteins (15 μg) were separated by 10% SDS-PAGE and analyzed by western blot using SIG1 antibody (SIG1); (**b**) Plants described above (CL) were dark-adapted for 18 h (LD) and re-illuminated with normal light (50 μmol photons m^−2^ s^−1^) for 1 h (LDL). Total proteins (10 μg) were separated by 10% SDS-PAGE and analyzed by western blot using SIG1 antibody (SIG1). In both panels, CBB-stained gels are shown as loading controls.

Because binding levels of SIG1 with target promoters decreased drastically under high-light stress, the amount and/or binding activity of SIG1 could be down-regulated by intense light. To assess this possibility, accumulation of SIG1 before and after high-light stress was examined. Surprisingly, the protein level of SIG1 slightly increased rather than decreased (Figure 4a). In addition, we examined SIG1 levels in dark-adapted and re-illuminated plants and found that SIG1 accumulation was almost constant in all light conditions (Figure 4b). These data suggest that the expression and activity of SIG1 could be regulated at translational and post-translational levels (see Discussion).

## 3. Discussion

Many papers have examined transcriptional regulation in chloroplasts, but ChIP analysis has not yet contributed substantially to this field, in spite of its powerful ability to detect *in vivo* binding patterns of transcription factors. Although it can detect both direct binding with DNA and indirect association alongside other factor(s), it still has an advantage in that it can monitor *in vivo* characteristics of the protein of interest. In this study, we introduced the ChIP system to the research of chloroplasts in the model plant *A. thaliana* to further extend the field’s approaches for understanding transcriptional regulation. Transcriptional regulation by SIG1, a sigma factor in *A. thaliana*, was demonstrated, and both specific binding of SIG1 to target promoters and dynamic changes in its binding pattern could be clearly detected. Based on our results and recent reports applying the ChIP-based method to maize, tobacco and wheat [30–32], we expect ChIP analysis will allow the detailed functional characterization of many types of transcriptional regulators in chloroplasts in the future.

The previous report demonstrated that SIG1 could accumulate cotyledon and true leaves of 12 days-old seedlings [18]. In this study, accumulation of SIG1 in *A. thaliana* seedlings gradually increased over the course of leaf development (Figure 1b), suggesting that SIG1 could be required to function more at later stage. A similar pattern has been observed in a monocot plant, maize (*Zea mays*) [33]. Expression of ZmSIG1 could be detected at greater levels in leaf tips (containing matured chloroplasts) but was not detectable in the leaf base (containing developing chloroplasts). Considering previous reports showing that SIG6 and SIG2 are required for transcriptional regulation mainly during early chloroplast development [22–26], some regulatory cascade(s) organized by multiple sigma factors could be formed during plant development.

In a previous study in rice (*Oryza sativa*), OsSIG1 could be disrupted by insertion of the Tos17 transposon, and the expressions of several chloroplast genes were markedly reduced [34]. Those genes included *psaAB*, *psbBT*, and *psbEFLJ*, which were also identified in this work as SIG1-bound promoters in *A. thaliana*. This consistency suggests that the role of SIG1 in chloroplast transcriptional regulation is similar in both monocot and dicot plants and that our ChIP-based method could successfully detect SIG1-target genes *in vivo*. In addition to the conserved genes discussed above, we identified other target genes, such as *rbcL* and *clpP*, which might be caused by evolutionary divergence. Previous studies demonstrated that transcription of *rbcL* could be mediated by SIG1 *in vitro* [35], and one of *clpP* promoter was recognized by PEP, especially in later stages of leaf development [36]; together these results suggest that SIG1 is involved in the transcription of these genes under specific conditions. Both genes are indispensable for normal chloroplast development; *rbcL* codes for a subunit of RUBISCO, and the essential role of the ClpP protein, a proteolytic subunit of the ATP-dependent Clp protease, has been confirmed in tobacco [37]. SIG1 of *A. thaliana* might have specific and significant roles related to transcription of these genes at any stage. Although a T-DNA insertion line for *sig1* was reported in *A. thaliana* [38], detailed molecular genetic analyses for AtSIG1 have not yet been performed. Thus, overall transcriptional regulation by SIG1 will be further clarified in the future.

Expression of *SIG1* mRNA was highly induced by light [12,13,17], but in this study, SIG1 protein levels were similar in both dark and light conditions (Figure 4b). In addition, the SIG1 protein accumulated to slightly higher levels under high-light stress (Figure 4a), while mRNA levels did not change in the presence of abiotic stresses [21]. Furthermore, the binding activity of SIG1 to its target promoters *in vivo* could be detected specifically under normal-light conditions (Figures 2 and 3). Taken together, this data suggested that expression of SIG1 could be regulated by some translational and/or post-translational mechanism(s). Recent studies demonstrated that chloroplast sigma factors, including SIG1, could be phosphorylated to adjust their activities in target gene transcription [39–41]. This type of post-translational modification(s) might be important to regulate the stability and/or activity of SIG1.

The expression of SIG5 was previously shown to be specifically induced under high-light stress to regulate transcription from a set of promoters, including *psbD* BLRP, which makes tolerance of various stresses possible [21]. In these conditions, SIG1 appears to be released to repress transcription of its target genes, while the SIG1 protein level was enhanced, suggesting that SIG1 may accumulate in preparation for rapid transcription initiation of target genes after rescue from the high-light condition. Based on this hypothesis, the detailed architecture of transcriptional regulation in chloroplasts will be investigated in the future, assisted by the use of novel techniques like ChIP analyses.

## 4. Experimental Section

### 4.1. Plant Materials and Growth Conditions

Seeds of *A. thaliana* ecotype Columbia (Col-0) were sterilized with 70% ethanol and 3% sodium hypochlorite before sowing on MS plates containing 0.4% Gelrite (Wako, Osaka, Japan) or Jiffy 7 (AS Jiffy Products, Norway). After stratification at 4 °C for 24 h in the dark, the seeds were grown at 23 °C under continuous white light (50 μmol photons m^−2^ s^−1^) and harvested after 1, 2, 3, or 4 weeks. For high-light stress treatment, the plants grown for four weeks were exposed to 1,200 μmol photons m^−2^ s^−1^ at 23 °C for 1 h. For light-dark treatment, the plants grown for four weeks were dark-adapted for 18 h and re-illuminated under same light conditions (50 μmol photons m^−2^ s^−1^) for 1 h.

### 4.2. Preparation of Polyclonal Antibody and Immunoblotting

The coding region for SIG1 was amplified from the *SIG1* full-length cDNA clone [13] with a primer pair (F: 5′-CACGACGTTGTAAAACGACATATGGCTACTGCAGCTGTTAT-3′ and R: 5′-GGATAACAATTTCACACAGGATCCGCTCTCTATGGCTCTG-3′) containing *Nde*I and *Bam*HI sites (underlined), respectively and was cloned into *Nde*I-*Bam*HI-digested pET15b (Merck Millipore, Darmstadt, Germany) to generate an in-frame fusion with the 6xHis-tag under the control of the T7 promoter. The recombinant SIG1 protein was expressed and purified in denatured condition using Ni-NTA agarose (Qiagen, Hilden, Germany) following the manufacturer’s protocol. The rabbit polyclonal antibody was prepared at RIKEN Bioresource Center. Extraction of total proteins and immunoblotting were performed as previously reported [22].

### 4.3. Preparation of Leaf Extracts for ChIP Analysis

Extracts from *A. thaliana* leaves were obtained as previously reported, with a few modifications [42]. Briefly, ~5 g of young leaves were harvested from four-week-old plants, washed briefly with Milli-Q water three times, and diced into ~10 mm^2^ pieces in ~100 mL of ice-cold TBS (20 mM Tris, pH 7.6, and 200 mM NaCl). Then, Silwet L-77 (NUC, Tokyo, Japan) was added to a final concentration of 0.01% (*v*/*v*) and mixed well. To crosslink DNA-protein complexes, formaldehyde was added immediately to a final concentration of 1% (*v*/*v*). Leaves were fixed by vacuum-infiltration (15 min × 2) at 700–800 mm Hg and washed (2 h × 2) with 150 mL of fresh TBS solution containing 0.3 M Glycine at 4 °C.

Leaves were filtered through two layers of Miracloth (Merck Millipore, Darmstadt, Germany) into a test tube and frozen in liquid nitrogen. They were ground to a fine powder in liquid nitrogen with a Multibeads Shocker (Yasui Kikai, Osaka, Japan) at 1800 rpm (30 s × 2). The powder was resuspended in 3 mL of lysis buffer (50 mM Hepes–KOH, pH 7.5, 140 mM NaCl, 1 mM EDTA, 1% Triton X-100, 0.1% sodium deoxycholate, Complete Mini protease inhibitor cocktails (Roche, Basel, Switzerland), and 10% glycerol) and homogenized by vortexing. The resultant slurry was filtered through two layers of Miracloth, and residual powder was washed with ~1 mL of lysis buffer and pooled. Crude extracts in the filtrate were sonicated on ice with a sonifier 250 (Branson, CT, USA) on setting 3 and 60% duty cycle (15 s × 10) with cooling on ice (>1 min) between sonication cycles.

The sample was then aliquoted into five eppendorf tubes (~800 μL each) and centrifuged at 15,000*g* for 20 min at 4 °C. The supernatant was further purified by centrifugation once more. Final (combined) supernatants were divided into aliquots (~400 μL), flash-frozen in liquid nitrogen, and stored at −80 °C. Protein extract concentrations were determined with the BCA protein assay system (Thermo Fisher Scientific, IL, USA).

### 4.4. ChIP and Quantitative PCR Analysis

ChIP and qPCR analyses were performed as described previously [43], except that we used Protein A sepharose beads (GE Healthcare, WI, USA) and 2 mg of extracted proteins for each ChIP assay. The sequences of primer pairs used in the qPCR analysis are listed in Table 1.

## 5. Conclusion

Our results indicated that the ChIP-based method established here for *A. thaliana* chloroplasts is very useful for the identification of direct-target genes for proteins of interest as well as dynamic changes in *in vivo* binding patterns. In particular, this method could be a powerful tool for the identification of target promoters for essential transcription factors. The results of this study also suggested that SIG1 could be released from target promoters in response to high-light stress, whereas the SIG1 protein still accumulated under the same condition, implying a complicated regulatory system in chloroplasts. Together with other approaches, ChIP methods could clarify these regulations.

## Figures and Tables

**Table 1:** Primers used for ChIP-qPCR analysis.

	Forward (5′ to 3′)	Reverse (5′ to 3′)
*psbKI*	TTGATCATTACATAGAAT	AACAAAAATTGGTGTTCT
*psbDC*	AATAAAATCAAAAATTTTG	AGCGATCCTCCTATTCA
*rbcL*	ATGAAAGAATATACAATAA	AAGTCCCTCCCTACAAG
*accD*	ATCCTTCTTTTCATTTAG	AGAGCTTCTGGCCTCTA
*petLG*	TGAATTGAGTTCTTTTTA	GAAGGGACTCAATAAAA
*psbBT*	TTGGTACTTATCGGATAT	GGAAATACCCCTTTATCA
*ycf2*	GCCAATTCCAATAGACTT	TGATTCCTCCTAAATTGC
*trnV-16S rRNA*	ATGGCTCGAATCCGTAGT	TCCCCCATCAAGAAATAG
*rpl32*	ATTATTTAAATGAGTACT	TCAAAAATGAAAAAAAAT
*ycf1*	TTTAATAGGGAACCTCAA	AAACCTCCCTTTTTTCTT
*rps15*	GATACCAATTATAGCGGA	AAAAAGAAATCCTTCCCC
*rps12-7*	GTATGGATATGTAAAATACA	TTGTAGGGTGGATCTCG
*trnI-rpl23*	ATCCCACTGAATTGAATTG	TTAGTGGGGATCCTCGT
*psbN*	TTTACCATATTCGGAATT	TATTATAGAATTGAAAGA
*clpP*	TAGTTTTATTCATTCTCT	GAAATGAAAAAAAAAGAG
*psbEFLJ*	ATTATGTAACACCCCATT	ACTGAACTCCAGATATTC
*atpB*	AGGTTTCAGTTAGTTGA	AATAAAAAAAATATGTTAAA
*ndhC*	CTATTAAGTAATAAGTGTA	AGACGAACTCCTATGAA
*psaAB*	CATAATAGATCCGAACACT	TGAGTCCTCCTCTTTCC
*trnEYD*	AATATAAAAAGAAAGTATAT	ATACTTGCTCAACCGC
*rpoBC*	TTCCAATTGAATATAGTC	CTTTTTTGAATTTCCCAT
*atpFA*	ATAAGTCTCATTATTATTA	ATAATCTCCTCTTCTAG
*psbA*	GTGGATTCGCTTCTAATT	GGTAAAATCCTTGGTTTA

## References

[1] Sugita M., Sugiura M. (1996). Regulation of gene expression in chloroplasts of higher plants. Plant Mol. Biol.

[2] Sato S., Nakamura Y., Kaneko T., Asamizu E., Tabata S. (1999). Complete structure of the chloroplast genome of *Arabidopsis thaliana*. DNA Res.

[3] Leister D. (2003). Chloroplast research in the genomic age. Trends Genet.

[4] Goldschmidt-Clermont M. (1998). Coordination of nuclear and chloroplast gene expression in plant cells. Int. Rev. Cytol.

[5] Hess W.R., Börner T. (1999). Organellar RNA polymerases of higher plants. Int. Rev. Cytol.

[6] Shiina T., Tsunoyama Y., Nakahira Y., Khan M.S. (2005). Plastid RNA polymerases, promoters, and transcription regulators in higher plants. Int. Rev. Cytol.

[7] Hedtke B., Börner T., Weihe A. (1997). Mitochondrial and chloroplast phage-type RNA polymerases in *Arabidopsis*. Science.

[8] Hajdukiewicz P.T., Allison L.A., Maliga P. (1997). The two RNA polymerases encoded by the nuclear and the plastid compartments transcribe distinct groups of genes in tobacco plastids. EMBO J.

[9] Allison L.A., Simon L.D., Maliga P. (1996). Deletion of *rpoB* reveals a second distinct transcription system in plastids of higher plants. EMBO J.

[10] De Santis-Maciossek G., Kofer W., Bock A., Schoch S., Maier R.M., Wanner G., Rudiger W., Koop H.U., Herrmann R.G. (1999). Targeted disruption of the plastid RNA polymerase genes *rpoA*, *B* and *C1*: molecular biology, biochemistry and ultrastructure. Plant J.

[11] Allison L.A. (2000). The role of sigma factors in plastid transcription. Biochimie.

[12] Isono K., Shimizu M., Yoshimoto K., Niwa Y., Satoh K., Yokota A., Kobayashi H. (1997). Leaf-specifically expressed genes for polypeptides destined for chloroplasts with domains of sigma70 factors of bacterial RNA polymerases in *Arabidopsis thaliana*. Proc. Natl. Acad. Sci. USA.

[13] Tanaka K., Tozawa Y., Mochizuki N., Shinozaki K., Nagatani A., Wakasa K., Takahashi H. (1997). Characterization of three cDNA species encoding plastid RNA polymerase sigma factors in *Arabidopsis thaliana*: Evidence for the sigma factor heterogeneity in higher plant plastids. FEBS Lett.

[14] Fujiwara M., Nagashima A., Kanamaru K., Tanaka K., Takahashi H. (2000). Three new nuclear genes, *sigD*, *sigE* and *sigF*, encoding putative plastid RNA polymerase sigma factors in *Arabidopsis thaliana*. FEBS Lett.

[15] Lysenko E.A. (2007). Plant sigma factors and their role in plastid transcription. Plant Cell Rep.

[16] Schweer J., Türkeri H., Kolpack A., Link G. (2010). Role and regulation of plastid sigma factors and their functional interactors during chloroplast transcription—Recent lessons from *Arabidopsis thaliana*. Eur. J. Cell Biol.

[17] Kestermann M., Neukirchen S., Kloppstech K., Link G. (1998). Sequence and expression characteristics of a nuclear-encoded chloroplast sigma factor from mustard (*Sinapis alba*). Nucleic Acids Res.

[18] Privat I., Hakimi M.A., Buhot L., Favory J.J., Lerbs-Mache S. (2003). Characterization of *Arabidopsis* plastid sigma-like transcription factors SIG1, SIG2 and SIG3. Plant Mol. Biol.

[19] Zghidi W., Merendino L., Cottet A., Mache R., Lerbs-Mache S. (2007). Nucleus-encoded plastid sigma factor SIG3 transcribes specifically the *psbN* gene in plastids. Nucleic Acids Res.

[20] Favory J.J., Kobayshi M., Tanaka K., Peltier G., Kreis M., Valay J.G., Lerbs-Mache S. (2005). Specific function of a plastid sigma factor for *ndhF* gene transcription. Nucleic Acids Res.

[21] Nagashima A., Hanaoka M., Shikanai T., Fujiwara M., Kanamaru K., Takahashi H., Tanaka K. (2004). The multiple-stress responsive plastid sigma factor, SIG5, directs activation of the *psbD* blue light-responsive promoter (BLRP) in *Arabidopsis thaliana*. Plant Cell Physiol.

[22] Kanamaru K., Nagashima A., Fujiwara M., Shimada H., Shirano Y., Nakabayashi K., Shibata D., Tanaka K., Takahashi H. (2001). An *Arabidopsis* sigma factor (SIG2)-dependent expression of plastid-encoded tRNAs in chloroplasts. Plant Cell Physiol.

[23] Ishizaki Y., Tsunoyama Y., Hatano K., Ando K., Kato K., Shinmyo A., Kobori M., Takeba G., Nakahira Y., Shiina T. (2005). A nuclear-encoded sigma factor, *Arabidopsis* SIG6, recognizes sigma-70 type chloroplast promoters and regulates early chloroplast development in cotyledons. Plant J.

[24] Hanaoka M., Kanamaru K., Takahashi H., Tanaka K. (2003). Molecular genetic analysis of chloroplast gene promoters dependent on SIG2, a nucleus-encoded sigma factor for the plastid-encoded RNA polymerase, in *Arabidopsis thaliana*. Nucleic Acids Res.

[25] Nagashima A., Hanaoka M., Motohashi R., Seki M., Shinozaki K., Kanamaru K., Takahashi H., Tanaka K. (2004). DNA microarray analysis of plastid gene expression in an *Arabidopsis* mutant deficient in a plastid transcription factor sigma, SIG2. Biosci. Biotechnol. Biochem.

[26] Loschelder H., Schweer J., Link B., Link G. (2006). Dual temporal role of plastid sigma factor 6 in *Arabidopsis* development. Plant Physiol.

[27] Tsunoyama Y., Ishizaki Y., Morikawa K., Kobori M., Nakahira Y., Takeba G., Toyoshima Y., Shiina T. (2004). Blue light-induced transcription of plastid-encoded *psbD* gene is mediated by a nuclear-encoded transcription initiation factor, AtSig5. Proc. Natl. Acad. Sci. USA.

[28] Aparicio O.M., Geisberg J.V., Struhl K, Ausubel F.A., Brent R., Kingston R.E., Moore D.D., Seidman J.G., Smith J.A., Struhl K. (2004). Chromatin Immunoprecipitation for Determining the Association of Proteins with Specific Genomic Sequences *in vivo*. Current Protocols in Molecular Biology.

[29] Kuo M.H., Allis C.D. (1999). *In vivo* cross-linking and immunoprecipitation for studying dynamic Protein: DNA associations in a chromatin environment. Methods.

[30] Prikryl J., Watkins K.P., Friso G., van Wijk K.J., Barkan A. (2008). A member of the Whirly family is a multifunctional RNA- and DNA-binding protein that is essential for chloroplast biogenesis. Nucleic Acids Res.

[31] Newell C.A., Gray J.C. (2010). Binding of *lac* repressor-GFP fusion protein to *lac* operator sites inserted in the tobacco chloroplast genome examined by chromatin immunoprecipitation. Nucleic Acids Res.

[32] Yagi Y., Ishizaki Y., Nakahira Y., Tozawa Y., Shiina T. (2012). Eukaryotic-type plastid nucleoid protein pTAC3 is essential for transcription by the bacterial-type plastid RNA polymerase. Proc. Natl. Acad. Sci. USA.

[33] Lahiri S.D., Allison L.A. (2000). Complementary expression of two plastid-localized sigma-like factors in maize. Plant Physiol.

[34] Tozawa Y., Teraishi M., Sasaki T., Sonoike K., Nishiyama Y., Itaya M., Miyao A., Hirochika H. (2007). The plastid sigma factor SIG1 maintains photosystem I activity via regulated expression of the *psaA* operon in rice chloroplasts. Plant J.

[35] Hakimi M.A., Privat I., Valay J.G., Lerbs-Mache S. (2000). Evolutionary conservation of *C*-terminal domains of primary sigma(70)-type transcription factors between plants and bacteria. J. Biol. Chem.

[36] Zoschke R., Liere K., Börner T. (2007). From seedling to mature plant: *Arabidopsis* plastidial genome copy number, RNA accumulation and transcription are differentially regulated during leaf development. Plant J.

[37] Shikanai T., Shimizu K., Ueda K., Nishimura Y., Kuroiwa T., Hashimoto T. (2001). The chloroplast *clpP* gene, encoding a proteolytic subunit of ATP-dependent protease, is indispensable for chloroplast development in tobacco. Plant Cell Physiol.

[38] Schweer J., Geimer S., Meurer J., Link G. (2009). *Arabidopsis* mutants carrying chimeric sigma factor genes reveal regulatory determinants for plastid gene expression. Plant Cell Physiol.

[39] Schweer J., Türkeri H., Link B., Link G. (2010). AtSIG6, a plastid sigma factor from *Arabidopsis*, reveals functional impact of cpCK2 phosphorylation. Plant J.

[40] Shimizu M., Kato H., Ogawa T., Kurachi A., Nakagawa Y., Kobayashi H. (2010). Sigma factor phosphorylation in the photosynthetic control of photosystem stoichiometry. Proc. Natl. Acad. Sci. USA.

[41] Türkeri H., Schweer J., Link G. (2012). Phylogenetic and functional features of the plastid transcription kinase cpCK2 from *Arabidopsis* signify a role of cysteinyl SH-groups in regulatory phosphorylation of plastid sigma factors. FEBS J.

[42] Johnson C., Boden E., Arias J. (2003). Salicylic acid and NPR1 induce the recruitment of trans-activating TGA factors to a defense gene promoter in *Arabidopsis*. Plant Cell.

[43] Hanaoka M., Tanaka K. (2008). Dynamics of RpaB-promoter interaction during high light stress, revealed by chromatin immunoprecipitation (ChIP) analysis in *Synechococcus elongatus* PCC 7942. Plant J.

